# Noninvasive, label-free, three-dimensional imaging of melanoma with confocal photothermal microscopy: Differentiate malignant melanoma from benign tumor tissue

**DOI:** 10.1038/srep30209

**Published:** 2016-07-22

**Authors:** Jinping He, Nan Wang, Hiromichi Tsurui, Masashi Kato, Machiko Iida, Takayoshi Kobayashi

**Affiliations:** 1 National Astronomical Observatories/Nanjing Institute of Astronomical Optics & Technology, Chinese Academy of Sciences, 188 Bancang Street, Nanjing, Jiangsu 210042, China; 2 Advanced Ultrafast Laser Research Center, University of Electro-Communications, 1-5-1 Chofugaoka, Chofu, Tokyo 182-8585, Japan; 3 JST, CREST, 5 Sanbancho, Chiyoda-ku, Tokyo 102-0075, Japan; 4 Department of Pathology, Juntendo University School of Medicine, Tokyo 113-8421, Japan; 5 Department of Occupational and Environmental Health, Graduate School of Medicine, Nagoya University, 65 Tsurumai-cho Showa-ku, Nagoya-shi, Aichi 466-8550, Japan; 6 Department of Electrophysics, National Chiao-Tung University, 1001 Ta Hsinchu Rd., Hsinchu 300, Taiwan; 7 Insitute of Laser Engineering, Osaka University, 2–6 Yamada-oka, Suita, Osaka 565-0971, Japan

## Abstract

Skin cancer is one of the most common cancers. Melanoma accounts for less than 2% of skin cancer cases but causes a large majority of skin cancer deaths. Early detection of malignant melanoma remains the key factor in saving lives. However, the melanoma diagnosis is still clinically challenging. Here, we developed a confocal photothermal microscope for noninvasive, label-free, three-dimensional imaging of melanoma. The axial resolution of confocal photothermal microscope is ~3 times higher than that of commonly used photothermal microscope. Three-dimensional microscopic distribution of melanin in pigmented lesions of mouse skin is obtained directly with this setup. Classic morphometric and fractal analysis of sixteen 3D images (eight for benign melanoma and eight for malignant) showed a capability of pathology of melanoma: melanin density and size become larger during the melanoma growth, and the melanin distribution also becomes more chaotic and unregulated. The results suggested new options for monitoring the melanoma growth and also for the melanoma diagnosis.

Skin cancer is one of the most common cancers. Melanoma accounts for less than 2% of skin cancer cases but causes a large majority of skin cancer deaths^1^. The rates of melanoma also have been growing for at least 30 years^2^. The most dangerous characteristic of melanoma is the capability of deep invasion, as it can spread over the body through lymphatic and blood vessels. For this reason, early detection and therapy of melanoma is of crucial importance in saving lives. Presently, the best method for clinical detection of melanoma is dermoscopy^3^. Based on the results of the Consensus Ne Meeting on Dermoscopy, the best sensitivity of dermoscopy is 83.7%, and the best specificity is 83.4%^4^. The reliability of this technique needs further improvement, and melanoma diagnosis is still clinically challenging. Since melanin carries the information about the metabolism and location of melanocytes and melanogenesis, the distribution of melanin could act as a marker for melanoma^5^,^6^. Two dominant types of melanin, eumelanin and pheomelanin, have large absorption of visible light without efficient fluorescence emission^7^, which makes it possible to image melanoma with photothermal (PT) microscopy (PTM).

PTM, which relies on the detection of local heating induced by sample’s optical absorption, has shown potential in biological imaging and clinical applications. The key advantages of PTM are high sensitivity and no requirement of staining^8^,^9^,^10^. It can image nanometer sized absorbers among scatters with high resolution, high signal-to-noise ratio (SNR) and in real time^11^,^12^,^13^. However, the PT signal in normal PTM (NPTM) has two extrema in axial direction^14^, which will introduce distortions and poor axial resolution to three-dimensional (3D) PT imaging. Confocal PTM (CPTM), which has a detection scheme similar to the confocal microscopy, can help to remove the drawback and improve the axial resolution^14^.

In this paper, we have developed a CPTM for noninvasive, label-free, 3D imaging of melanoma. The performance of the setup is tested with a sample of 20-nm gold nanoparticle. An axial resolution enhancement of ~3 times is achieved compared with NPTM. Then, 3D microscopic distributions of melanin in benign and malignant melanoma tissue are obtained with this setup. The statistic discussions of sixteen 3D images showed marked differences in the density and shapes of melanin for the benign and malignant tissues. The 3D fractal analysis of all the images is also performed, and the malignant melanoma has a larger fractal dimension. The detection of melanin distributions in melanoma using CPTM can be a new option for melanoma diagnosis.

## Experimental setup

Supplementary Fig. S1 outlines the experimental setup. The pump and probe beams, with central wavelength of 488 and 632.8 nm, respectively, are spectrally filtered from a compact supercontinuum fiber laser source (WL-SC450-2, 20 MHz, Fianium, UK) with bandpass filters (FL488-10, FL632.8-10, Thorlabs). Considering the light absorption coefficient of melanin and other molecules in skin^15^ and the optical spectral density of the fiber laser source, the center wavelength of 488 nm seems to be the best choice for the pump beam in our experiment. The powers of pump and probe are 0.43 mW and 0.28 mW, respectively. The intensity of the pump pulse is modulated at 30 kHz with an electro-optic modulator (EOM) (LM202P, Qioptiq, Germany). Two sets of lenses are used to expand the pump and probe beams and adjust the divergence of the two beams. An objective lens (60×/NA 0.9, UPlanFLN, Olympus) is used to focus the two beams into the specimen. The 3D scanning of the samples is performed with a set of piezo stages (PS) (P-622.2CL and P-622.ZCL, Physik Instrumente (PI), Germany).

The detection module can be divided into three parts (see Detections 1–3 in Fig. S1). Detection 1 is for the optimization of the axial overlapping of pump and probe beams. The back scattered pump and probe beams from the sample (silver film) are focused into a fiber with a diameter of 25 μm by an achromatic lens (AC254-100-A, Thorlabs) with focal length of 100 mm. The detector in Detection 1 is the CCD camera in a spectrometer (USB 4000, Ocean Optics), which can accumulate both the pump and probe simultaneously together with our home-made data acquisition and processing software. The axial overlapping of the pump and probe is optimized by adjusting the beam divergence of the pump and probe.

**Figure 1:**
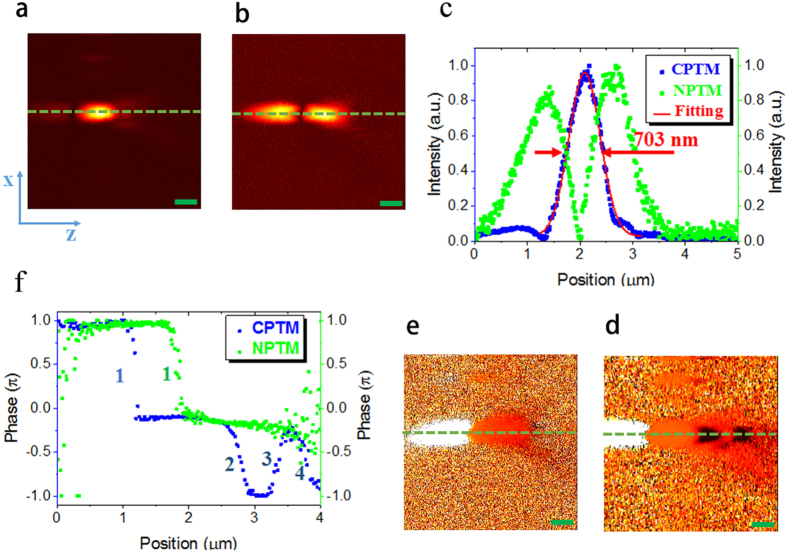
Intensity (**a**,**b**) and phase (**d**,**e**) distributions in XZ plane for the PT signal obtained with CPTM (**a**,**d**) and NPTM (**b**,**e**). The sample is a single 20-nm GNP. Scale bar: 500 nm. The cross sections (denoted by the red dashed lines in (**a**,**b**,**d**,**e)**) of the intensity image (**a**,**b**) and phase image (**d**,**e**) of the GNP are shown in (**c**,**f**) respectively. The axial resolution of CPTM is 703 nm, and that for NPTM is worse than 2 μm.

The forward propagating beams are collected and collimated by a condenser lens (100×/NA 1.4, Olympus). After passing through the condenser lens, the probe beam is spectrally filtered out by a band pass filter (FL632.8-10, Thorlabs). Then, the probe beam is split into two by a beam splitter (BSW26R, Thorlabs) for Detections 2 and 3, which are used respectively for NPTM and CPTM. The aperture of the iris in Detection 2 are optimized to obtain the maximum PT signal, while the position of the fiber in Detection 3 is optimized to minimize the side lobe intensity (see in Fig. 1(c)). The focal length of the lens (AC254-100-A, Thorlabs) in Detection 3 is 100 mm. In the section of Test of axial properties of CPTM, the two detection modules are applied simultaneously. While in the section of 3D imaging of benign and malignant melanoma with CPTM, only Detection 3 is used in the experiment. The detectors in Detection 2 and Detection 3 are two auto-balanced detectors (BD) (Nirvana 2007, Newport), which can help to reduce the laser noise of probe beam by ~20 dB. The PT signals are demodulated by two lock-in amplifiers (LIA1, 2) (SR844, Stanford Research System, US; Model 7265, Signal Recovery, US). An AD/DA converter is applied to transfer the signals from LIAs to computer (PC) and send the command from the PC to the PS.

Three types of samples are used in the research. A sample of silver film is used to calibrate the axial overlapping of pump and probe; a sample of 20-nm gold nanoparticle is used to test the axial properties of CPTM and NPTM; two malignant melanoma samples (MMS1 and MMS2) and one benign melanoma sample (BMS1) are used in the biomedical imaging study, and the main object of the study is to differentiate the malignant melanoma from the benign tumor tissue with our home-made CPTM.

The sample of silver film is grown on a slide glass (Matsunami, Japan) with a CVD method by Dr. Nakata (University of Electro & Communications, Japan). The size of the silver film is 2 mm × 6 mm × 0.01 mm.

Gold nanoparticles (GNP) of 20-nm diameter stabilized suspension in 0.1 mM PBS with optical density of 1.0 is supplied by Sigma Aldrich. A drop of such suspension is diluted by 100 times with distilled water, and then a drop of the diluted suspension is spread on the slide glasses (Matsunami, Japan). After the suspension become dried, a piece of cover glass (Matsunami, Japan) is applied to cover the sample.

RET-mice that are introduced an oncogene RET (RFP-RET) under metallothionein-I promoter enhancer spontaneously develop benign melanocytic tumor and melanoma. Macroscopic and microscopic appearances of a benign melanocytic tumor and melanoma were shown in our previous reports^16^,^17^ (Fig. 3 in ref. 16 and Fig. 1 in ref. 17). Since both benign melanocytic tumors and melanoma in RET-mice are usually developed as a hemispherical shape, there is no sampling direction. The samples of both benign or malignant melanoma were fixed with 3% buffered formalin, embedded in paraffin, sliced at 10–15 μm thick, spread on slide glasses and sealed with mounting medium. Two malignant melanoma samples (MMS1 and MMS2) and one benign melanoma sample (BMS1) are used in the study. All the experiments have been reviewed and approved by the Institutional Animal Care and Use Committee, Juntendo University School of Medicine with the approval #270108. The procedures using the animals were conducted according to the institutional guidelines.

## Results

### Test of the axial properties of CPTM

The axial overlapping of the pump and probe is firstly optimized by confocal scattering microscope with a sample of silver film. The setup of the confocal scattering microscope is a part of the whole setup as depicted in Fig. S1. The results of the axial overlapping of pump and probe are shown in Fig. S2. After the optimization, the axial offset of pump and probe is reduced from 140 nm (Fig. S2(a)) to <20 nm (Fig. S2(b)). The excellent axial overlapping of pump and probe will help to enhance the signal intensity^14^ and also the lateral resolution^18^ for CPTM. Then, the axial distributions of the point spread functions (PSFs) of CPTM and NPTM are tested with a sample of 20-nm GNP. Figure 1 depicts an XZ (Z axis is in the axial direction) scan of a single GNP with CPTM and NPTM. The PT signal for NPTM has two extrema in axial direction (see Fig. 1(b)), and resulting in the full width at half maximum (FWHM) of the intensity distribution of ~2.2 μm (see green curve in Fig. 1(c). Compared with the case of NPTM, the PT signal obtained with CPTM has only a single peak in axial direction (Fig. 1(a)), with an enhanced axial resolution defined by FWHM of ~700 nm (blue and red curves in Fig. 1(c)) and an improvement of ~3 times. The phases of the PT signals obtained with CPTM and NPTM are shown in Fig. 1(d,e), respectively. The PT signal phase experiences 4-sharp jumps in the CPTM image, as depicted in Fig. 1(d) and the blue curve in Fig. 1(f). The four jumps, separating the two phase states (0 and π/−π), are corresponding to the four peaks (one main peak and three small side lobes, blue curve in Fig. 1(c)) in the intensity distribution profile, and the phase of the most intense peak is around 0. The phases of the two intensity peaks (green curve in Fig. 1(c)) for the case of NPTM have a difference of π, and only one phase jump exists in the whole scan range, as shown in Fig. 1(f) (green curve).

### 3D imaging of benign and malignant melanoma with CPTM

**Figure 2:**
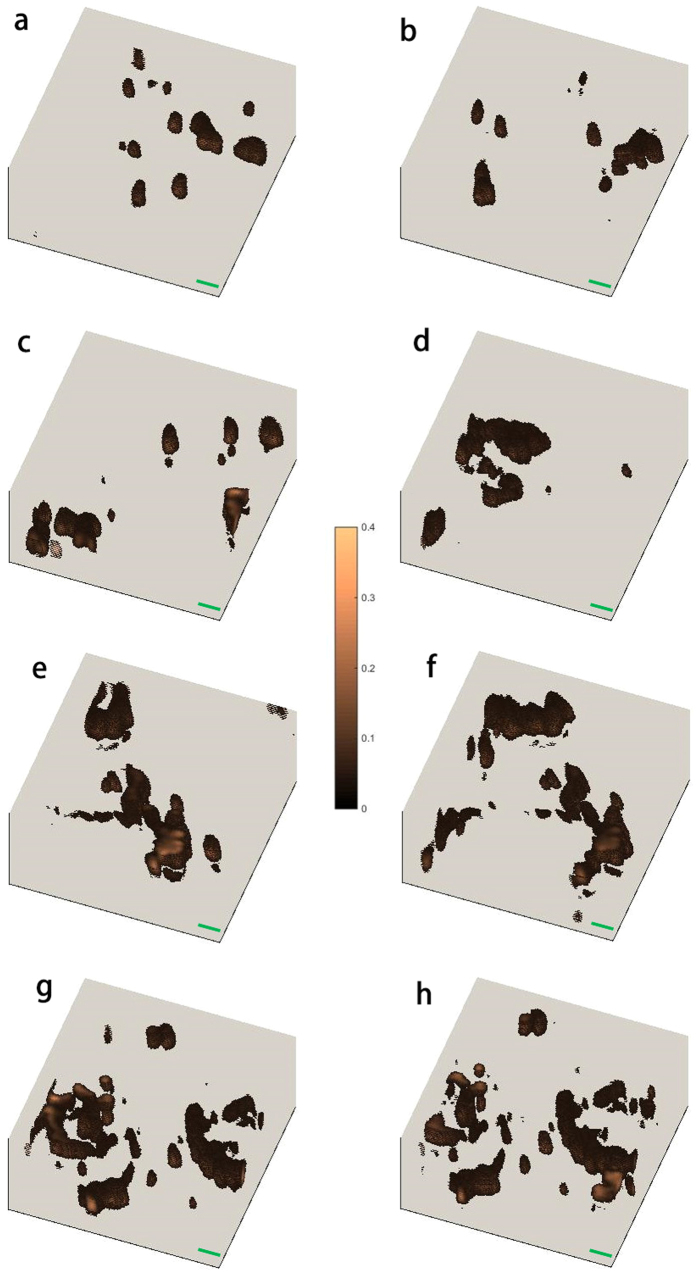
3D distributions of melanin in benign melanoma. **(a**–**f)** denote the 3D images of benign melanoma at different positions of the sample, with the images named B1–B8, respectively. Scale bar: 2 μm. Pump: 488 nm/0.43 mW/20 MHz/~100 ps. Probe: 632.8 nm/0.28 mW/20 MHz/~100 ps. Dwell time: 3 ms.

**Figure 3:**
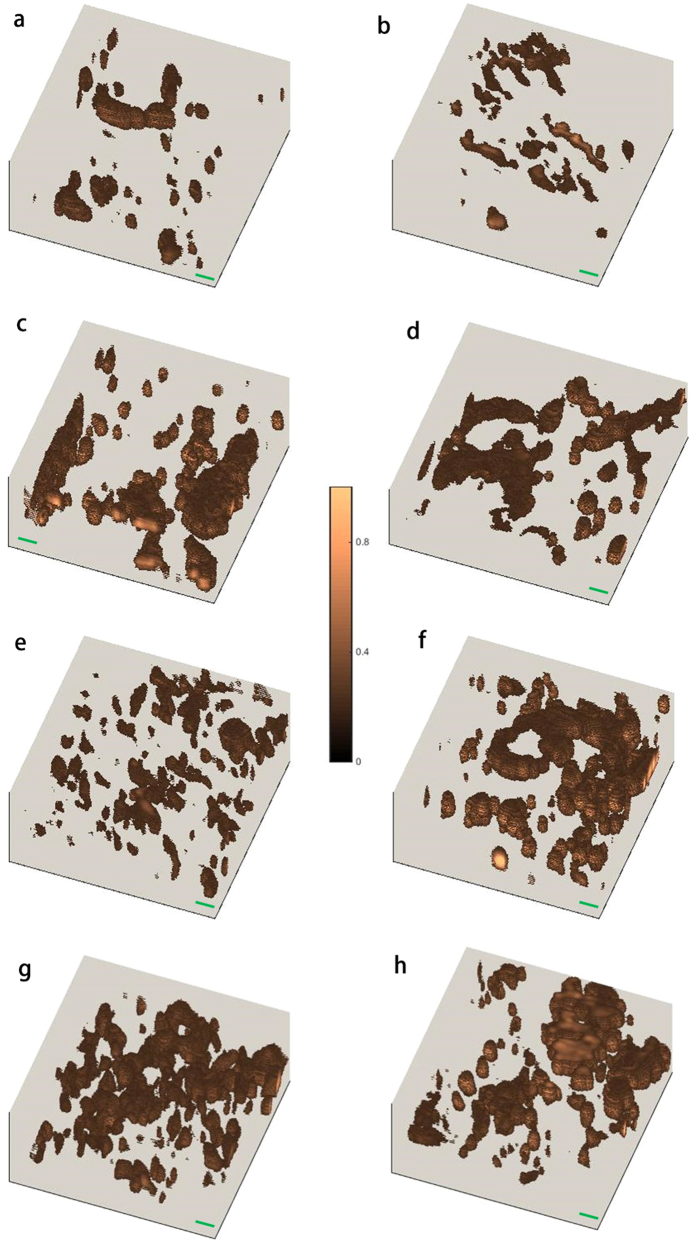
3D distributions of melanin in malignant melanoma. **(a**–**f)** denote the 3D images of benign melanoma at different positions of the sample, with the images named M1–M8, respectively. Scale bar: 2 μm. Pump: 488 nm/0.43 mW/20 MHz/~100 ps. Probe: 632.8 nm/0.28 mW/20 MHz/~100 ps. Dwell time: 3 ms.

After the optimization and calibration of the setup, the home-made CPTM is applied to the 3D imaging of mouse melanoma without any staining. To maintain high SNR images, the time constant of the lock-in amplifier (LIA) and the dwell time of the imaging are both set as 3 ms. Under this condition, the output voltage of the maximum PT signals are typically in the range of 0.5–2 V and the noise level of all the images is ~0.05 V with the SNR of higher than 10. To perform the statistical analysis of melanoma, especially to demonstrate the differences between benign and malignant melanoma, we have performed eight 3D scans at different positions of the samples for both the benign and malignant melanoma. The range of the 3D images is 22.5 μm × 22.5 μm × 11.25 μm, with the whole pixel number of 200 × 200 × 50. The 3D images for benign and malignant melanoma are depicted in Figs 2 and 3, respectively. The brown dots or aggregations in these images are melanin, which shows the metabolism and location of melanocytes and melanogenesis. The gray background is the skin tissue, which induces much lower PT signal because of its much lower absorption of pump light compared with melanin^15^. Some other molecules or organelles in the skin, such as hemoglobin, also absorb pump light and induce PT signal. However, because the absorption coefficient of hemoglobin is at least 3 times smaller than that of melanin in the case of the pump wavelength of 488 nm^15^, the PT signal generated from hemoglobin also should be smaller than that generated from melanin. Figure S3 depicts the 3D imaging of benign melanoma tissue with very low melanin density, and from this figure we can find some structures of organelles, which may be hemoglobin. The PT signal of such structures is just above the noise level, and it is typically 10–20 times smaller than the PT signal generated from melanin. The high contrast of the imaging of melanin in the skin tissue is possible by careful choice of pump wavelength. Utilizing the sixteen 3D images shown in Figs 2 and 3, we discuss melanoma pathology based on classic morphometric methods (size, shape, density…) and fractal analysis.

### Pathology of melanoma: melanin density

**Figure 4:**
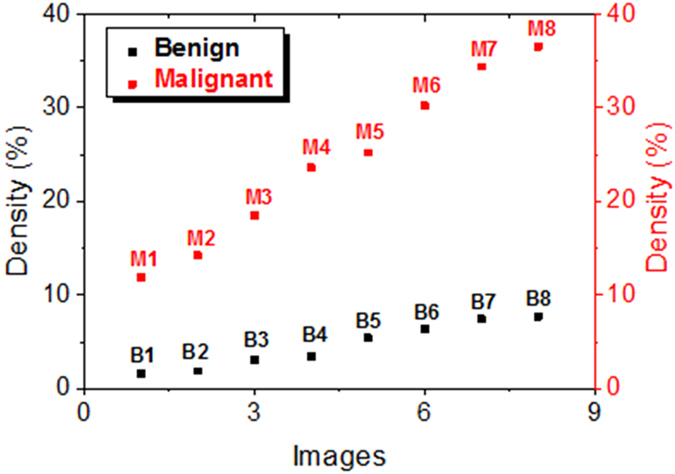
Melanin density of benign and malignant melanoma plotted in the order of the increasing densities of benign and malignant samples.

From Figs 2 and 3, we can find that the melanin density is lower for benign melanoma compared with malignant one intuitively. However, to demonstrate the difference quantitatively, we firstly calculated the melanin density in the sixteen 3D images in Figs 2 and 3. The method for the melanin density calculation is demonstrated in the section Methods. Figure 4 depicts the melanin density of the sixteen 3D images (eight for benign melanoma B1–B8, and eight for malignant melanoma M1–M8). We can find that the melanin densities of the eight benign melanoma images are all lower than 10%, with the maximum value of 7.7% (B8) and the minimum value of 1.6% (B1), seeing the black squares in Fig. 4. The average density of the eight benign melanoma images (B1–B8) is calculated to be 4.6% ± 2.4%. The melanin densities in malignant melanoma tissues are all higher than 10%, with the minimum value of 11.9% (M1) and the maximum value of 34.4% (M8). The average melanin density of the eight malignant melanoma images (M1–M8) is calculated to be 24.3 ± 9.1%, which is ~5 times larger than that of the benign melanoma samples. However, all the sixteen 3D images are taken in or near the center part (defined by the part with most intense PT signal) of the samples. The melanin density will be much lower in the periphery of the samples, for example, the melanin density can be nearly zero at the edge of the benign melanoma tissue. Based on the results in Fig. 4, we can use a melanin density of ~10% to differentiate the benign and malignant melanoma. The melanocytes are only concentrated in several localized positions for the benign melanoma tissues. And the melanin, which represents the melanocytes, has spread over and also becomes denser when the melanoma becomes malignant.

### Pathology of melanoma: size distribution of melanin particles/aggregations

**Figure 5:**
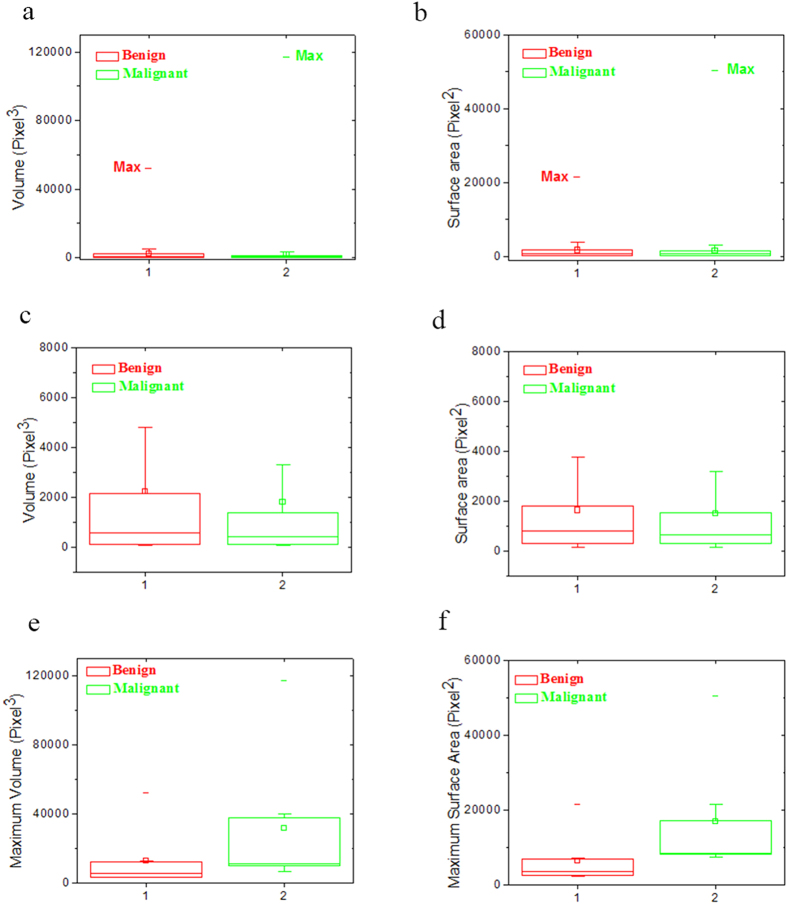
Box plots of the size information of melanin particles/aggregations in benign and malignant melanoma. (**a**,**b**) are box plots of volume and surface area, respectively, of the melanin particles/aggregations in the sixteen 3D images in Figs 2 and 3. (**c**) The box plot of volume with small plot range compared with (**a**). (**d**) The box plot of surface area with small plot range compared with (**b**). (**e**,**f**) are box plots of the volumes and surface areas of the maximum melanin particles/aggregations in each image in Figs 2 and 3.

Besides the difference in the melanin density, the size distribution of melanin particles/aggregations in benign and malignant melanoma also shows some difference, as depicted in Figs 2 and 3. To give a quantitative discussion, we have calculated the melanin size distributions of melanin particles/aggregations in the sixteen 3D images in Figs 2 and 3. The size information, such as one dimensional (1D) (distance and length), two dimensional (2D) (surface area) and 3D (volume) information, can be calculated with the software ImageJ, and the detailed information is given in Methods section. We only demonstrate and discuss the surface area and volume in the present work. During the calculation and discussion, units of pixel^3^ and pixel^2^ are used for volume and surface area, respectively, with the pixel unit of 112.5 nm. The total numbers of the melanin particles/aggregations in malignant melanoma images (M1–M8) and benign melanoma images (B1–B8) are calculated to be 319 and 105, respectively. In order to depict the statistics of the 2D and 3D size data of all the melanin particles/ aggregations graphically, box plot is applied. Conventionally, five values from a set of data are used in box plot: the extremes, the upper and lower quartiles, and the median^19^. Figure 5(a,b) show the box plots of volumes and surface areas of the melanin particles/aggregations in the sixteen 3D images in Figs 2 and 3. As shown by the upper datum (denoted by the word “Max”) in Fig. 5(a,b), the maximum melanin aggregation in benign melanoma, has a volume of 52020 pixel^3^ (~74 μm^3^) and a surface area of 21561 pixel^2^ (273 μm^2^), while the volume and surface area of the maximum melanin aggregation in malignant melanoma are calculated to be 116944 pixel^3^ (~167 μm^3^) and 50424 pixel^2^ (638 μm^2^), respectively. The largest melanin aggregation in malignant melanoma is ~2 times larger in size than that in the benign melanoma. In order to show the box, which is formed by the first and third quartiles, more clearly, we have reduced the ranges of the box plots, and the plots are depicted in Fig. 5(c,d). The mean sizes (both volume and surface area) of the melanin particles/aggregations, denoted by the small squares in Fig. 5(c,d), show a difference of ~10–20% for benign and malignant melanoma. The widths of the boxes in the box plots of malignant melanoma images seem narrower than those in benign images, which means the sizes of the particles/aggregations in malignant melanoma are concentrated in a smaller range. However, more data is needed to justify whether the two parameters are effective criterions for differentiating malignant melanoma from benign one. Besides the box plots of the size data of all the melanin particles/aggregations in the sixteen 3D images in Figs 2 and 3, the box plots of the volumes and surface areas of the maximum melanin particles/aggregations in each image are also given, and the plots are depicted in Fig. 5(e,f). From the plots in Fig. 5(e,f), we find that the maximum values, the first and third quartiles, and the median of the size data for the eight particles/aggregations selected from malignant melanoma are all ~ 2 times of the corresponding values for the eight maximum particles/aggregations selected from the eight benign images, which means that the particles/aggregations grow during the melanoma progression process.

### Pathology of melanoma: Fractal dimension

**Figure 6:**
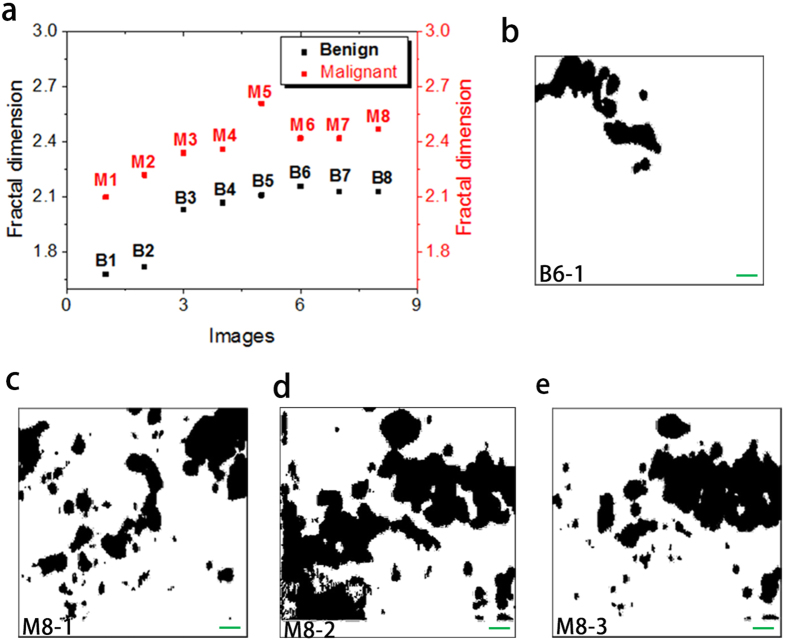
Fractal analysis of benign and malignant melanoma. (**a**) Fractal dimensions of all the sixteen 3D images 2D binary images from B6 (**b**) and M8 (**c**–**e**) are used for 2D analysis. The range of the 2D images are 22.5 μm × 22.5 μm. Scale bar: 2 μm. The fractal dimensions are 1.61, 1.56, 1.72 and 1.61 for (**b**–**d**,**f**) respectively.

The growth of cancer, including melanoma, is usually a chaotic and irregular process, and fractal dimension is well suited to describe the malignancies in a qualitative sense^20^,^21^,^22^,^23^,^24^,^25^. The raw images of cancer for fractal analysis can be obtained with fluorescence microscopy^20^, dermoscopy^26^ and atomic force microscopy^22^, but those images are typically 2D^20^,^21^,^23^,^24^,^25^ or pseudo-3D^22^ images at best. In the case of melanoma, several factors (organ or tissue) can be used in the fractal analysis, such as boundary between cancer and the host^20^,^26^, and nuclear chromatin^22^. Here, we performed the fractal analysis of benign and malignant melanoma based on 3D distributions of melanin, and the boundary between the melanin (the melanocytes) and the host (skin tissue) plays a key role in the analysis. The fractal dimensions of all the sixteen 3D images are calculated with the software ImageJ, and the details of the calculation are shown in the section of Methods. Figure 6(a) shows the fractal dimensions of all the sixteen 3D images in Figs 2 and 3. The fractal dimensions for benign melanoma (Black squares in Fig. 6(a)) are between 1.68 and 2.16, with the mean value of 2.00 and standard deviation of 0.19, whereas those for malignant melanoma (Red squares in Fig. 6(a)) are in the range of 2.10–2.61, with the mean value of 2.37 and standard deviation of 0.16. The malignant melanoma seems to be of larger fractal dimensions compared with the benign one, which means that the melanin distribution becomes more chaotic and poorly regulated during the melanoma cancer growth. Although better understanding of the melanin morphology in melanoma still need to be revealed by molecular methods^27^. To compare the reliability of 2D and 3D fractal analysis in melanoma diagnosis, we also have performed the fractal analysis of several 2D images for both benign and malignant melanoma. We chose one binary 2D image from B6 (B6-1, depth of 9.9 μm) and three binary 2D images from M8 (depth of 3.375 μm, 9 μm and 10.125 μm for M8-1, M8-2 and M8-3, respectively), as depicted in Fig. 6(b–e). The fractal dimension of B6-1 is calculated to be 1.61, while those of M8-1, M8-2 and M8-3 are 1.56, 1.72 and 1.61, respectively. Since the 2D images from different depths are very different among them, 2D fractal analysis is no longer suitable for differential benign melanoma from malignant one.

## Discussion

In the present work, we have obtained high resolution 3D images of benign and malignant melanoma with CPTM. Based on the sixteen captured 3D images (eight for benign and the other for malignant melanoma), we have demonstrated an understanding of melanoma pathology using classical morphometric methods (melanin density and size distribution) and fractal analysis. The melanin density in malignant melanoma is as high as 24.3%, and it is ~5 times of the value in benign melanoma. The maximum melanin aggregation in malignant melanoma has a volume of ~74 μm^3^, which is ~2 times larger than that in benign case. Based on the calculated results, we can conclude that the melanin, which represents the melanocytes, becomes denser and more aggregated when the melanoma becomes malignant. The fractal dimensions of benign and malignant melanoma are 2.0 and 2.4, respectively, which indicates that the melanin distributions become more chaotic and poorly organized during the growth of melanoma cancer. In comparison to the 2D fractal analysis, 3D fractal analysis is more useful and reliable in melanoma diagnosis.

Besides the well-established clinical melanoma diagnosis method—dermoscopy^3^,^26^,^28^, several other optical methods also show potentials to be used in melanoma diagnosis, such as confocal scanning laser microscopy (CSLM)^29^,^30^, optical coherent tomography (OCT)^31^. pump-probe microscopy (PPM)^6^ and photoacoustic microscopy (PAM)^32^,^33^. The main disadvantages of dermoscopy is relatively low sensitivity and lack of deep imaging capability of suspicious pigmented lesion^3^,^26^,^28^. Based on the summarization by D. S. Rigel^3^, CSLM can measure into papillary dermis with long wave length pump light, but it gives poor resolution of chromatin patterns, nuclear contours and nucleoli; OCT can give higher detection depth and resolution, but it is apt to image artifacts, and special materials are needed to reduce the scattering and increase the imaging depth. PPM can differentiate eumelanin and pheomelanin in pigmented lesions, however, the demanding of two femtosecond laser source will lead to high cost and large setup^6^. PAM can detect deep tumor with relative high resolution, although it typically needs the staining of metal nanoparticle to enhance the imaging contrast^31^,^32^. The method demonstrated in the present work is based on CPTM, which is a pure optical process. It can supply us with an imaging sensitivity of single molecule level^8^. The absorption coefficient of melanin is ten times larger than hemoglobin and other molecules in skin tissues even with pump wavelength in near infrared range^15^, it means that the label free imaging of melanoma with high contrast and great depth is feasible with the present method. Continuous-wave (CW) laser sources, such as diode lasers, can be used as the pump and probe beams for CPTM^18^, and it will make the imaging system very cheap and compact. The spatial resolution of CPTM is also better than PAM since CPTM is a PPM process. The advantages of the present method can be summarized as: 1) label-free; 2) no need of femtosecond lasers; 3) high spatial resolution; 4) 3D capacity; 5) high contrast; 6) high sensitivity. And these advantages make the present method a new option for monitoring the melanoma growth and also the melanoma diagnosis in clinics. The CPTM can also be converted or coupled to a photothermal cancer therapy system^34^,^35^ by just increasing the pump energy. However, due to two limitations, the experimental setup used to demonstrate the present method is not feasible for *in vivo* imaging of melanoma in the present phase. The first limitation is the imaging speed. Due to the high laser noise level and low average power of pump beam^36^, the imaging dwell time is set as 3 ms to obtain high SNR images. As a result, the imaging time for one 3D image is about 100 min, which is too slow for *in vivo* applications. To solve this problem, we are going to use high power stable CW laser source as the pump and probe beams. Typically, the stability of CW laser source can be better than 1%, and it is >10 times better than the beams spectrally filtered from SC laser source. It means the imaging dwell time can be reduced by >10 times without influencing the SNR much. The utilization of higher power pump and probe can also help to increase the SNR. The second limitation of the current setup is that the home-made sample stage is not ready for live animals currently. We are still proceeding the research to *in vivo* imaging of human melanoma.

## Methods

### CPTM

CPTM has been demonstrated both theoretically and experimentally by J. Moreau^14^,^37^. The probe beam spot size change on the pinhole (A single mode fiber in the present setup) plane, which can be treated as the photothermal signal, is given by^37^:


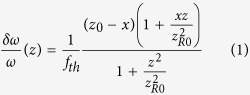


where *f*_*th*_ is the focal length of the thermal lens, *z*_0_ is the distance between the probe beam waist and the thermal lens, *z*_*R0*_ is the probe beam Rayleigh range in the sample. In the equation, *x* = (*z*_1 _+ *z*_2_ − *z*_1_*z*_2_/*f*  )/(1 − z_2_/*f*  ), with *f* the focal length of the focal lens for the detection, *z*_1_ the distance between the probe beam waist (in the sample) and the focal lens, *z*_2_ the distance between the focal lens and the pinhole. The confocal case corresponds to *x*/*z*_*R*0_ = ±1, while the standard non-confocal case corresponds to *x* → ∞.

### Evaluation of the melanin density in the melanoma tissue

Firstly, the signal intensity distributions of the 3D images are calculated with a homemade Matlab (MathWorks, US) program, and the results are shown in Figs S4 and S5. Then, we can set a critical signal intensity *I*_*c*_ to judge whether the current pixel is filled by melanin or not. The pixel with PT signal higher than *I*_*c*_ will be considered to be filled with melanin. Then the melanin density of the 3D images can be calculated with the expression *r* = *N*_*c*_/*N*_*t*_, where *N*_*c*_ is the number of the pixels with signal intensity higher than *I*_*c*_, *N*_*t*_ is the total pixel number of the 3D image. In the paper, *I*_*c*_ is set at 0.05 V, which is the noise level of the imaging system.

### The size distribution of melanin particles/aggregations in the melanoma tissue sample

The size distribution of melanin particles/aggregations is calculated with the software ImageJ (Fuji Is Just). The 2D 8-bit gray-type image sequence was firstly imported into the software ImageJ (Fuji Is Just), and then 3D images were reconstructed with the plugin ‘3D Viewer’. After that, we performed the statistics of the particle size (volume and surface area) distribution with ‘3D Objects Counter’ in the ‘Analyze’. The threshold for the calculation is set to be 10. Considering the real spatial resolution of the system and the simplification of the analysis, the size filter is set as 50 pixel^3^ (0.07 μm^3^). Then, the particle number, the maximum and mean values of the volume and surface area of the melanin in all the 3D images were obtained.

### Fractal analysis of benign and malignant melanoma

The fractal dimensions of all the 3D images are calculated with the software ImageJ (Fuji Is Just). The 2D image sequence is firstly imported into the software ImageJ, and the type of the 2D images is changed to 8-bit gray. Then the fractal dimension is calculated with plugin ‘FractalCount’. The parameters in the calculation are shown as Table S1 in the Supplement information. During the 2D fractal analysis, the images are firstly converted into binary images, and then the plugin ‘FractalCount’ is used to calculate the fractal dimension. The parameters for the 2D fractal analysis are also same as those in Table S1.

## Additional Information

**How to cite this article**: He, J. *et al*. Noninvasive, label-free, three-dimensional imaging of melanoma with confocal photothermal microscopy: Differentiate malignant melanoma from benign tumor tissue. *Sci. Rep*. **6**, 30209; doi: 10.1038/srep30209 (2016).

## Supplementary Material

Supplementary Information
