# Supplementary material for: Noninvasive, label-free, three-dimensional imaging of melanoma with confocal photothermal microscopy: Differentiate malignant melanoma from benign tumor tissue
Source: Sci Rep. 2016 Jul 22;6:30209. doi: 10.1038/srep30209 (PMC4957150; doi:10.1038/srep30209)
Supplement: Supplementary Information [file srep30209-s1.pdf]

Noninvasive, label-free, three-dimensional imaging of melanoma with confocal photothermal microscopy: Differentiate malignant melanoma from benign tumor tissue

Jinping He,[1,2,3] Nan wang,[2,3] Hiromichi Tsurui, [4] Masashi Kato,[5] Machiko Iida,[5] and Takayoshi Kobayashi[2,3,6,7,*]

[1]*National Astronomical Observatories/Nanjing Institute of Astronomical Optics & Technology, Chinese Academy of Sciences, 188 Bancang Street, Nanjing, Jiangsu 210042, China*

[2]*Advanced Ultrafast Laser Research Center, University of Electro-Communications, 1-5-1 Chofugaoka, Chofu, Tokyo 182-8585, Japan*

[3]*JST, CREST, 5 Sanbancho, Chiyoda-ku, Tokyo 102-0075, Japan*

[4] *Department of Pathology, Juntendo University School of Medicine, Tokyo 113-8421, Japan*

[5]*Department of Occupational and Environmental Health, Graduate School of Medicine, Nagoya University, 65 Tsurumai-cho Showa-ku, Nagoya-shi, Aichi 466-8550, Japan*

[6]*Department of Electrophysics, National Chiao-Tung University, 1001 Ta Hsinchu Rd., Hsinchu 300, Taiwan*

[7]*Insitute of Laser Engineering, Osaka University, 2-6 Yamada-oka, Suita, Osaka 565-0971, Japan*

***** Email: kobayashi@ils.uec.ac.jp.**

Supplementary information

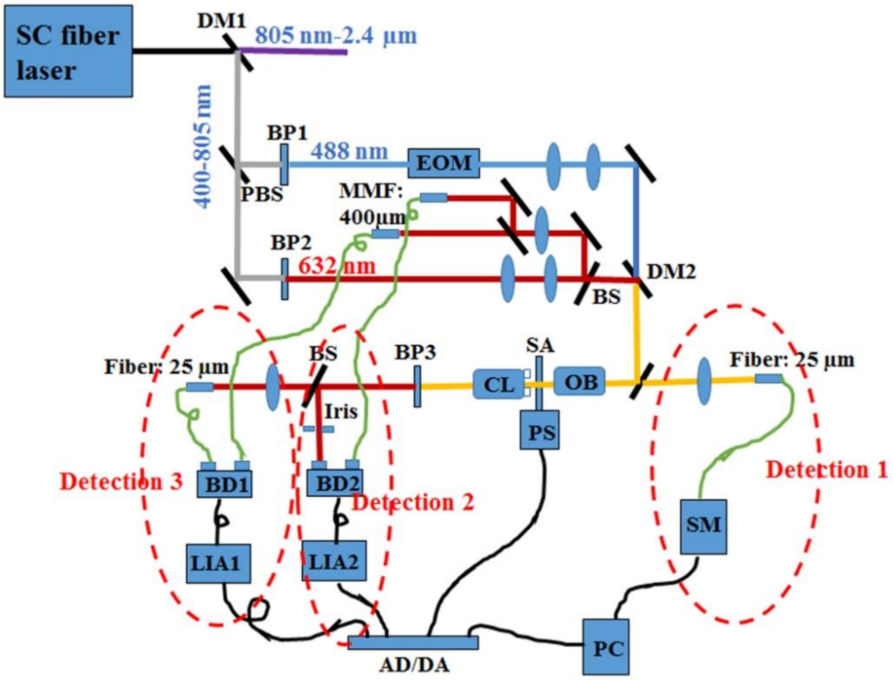

Figure S1 Schematic diagram of the imaging system. DM1, 2: dichroic mirrors; PBS: polarization beamsplitter; BP1, 2, 3: bandpass filter; EOM: electro-optic modulator; BS: beamsplitter; MMF: multimode fiber; OB: objective lens; PS: piezo stage; SA: samples; CL: condenser lens; BD: auto-balanced detector; LIA1, 2: lock-in amplifiers; SM: spectrometer

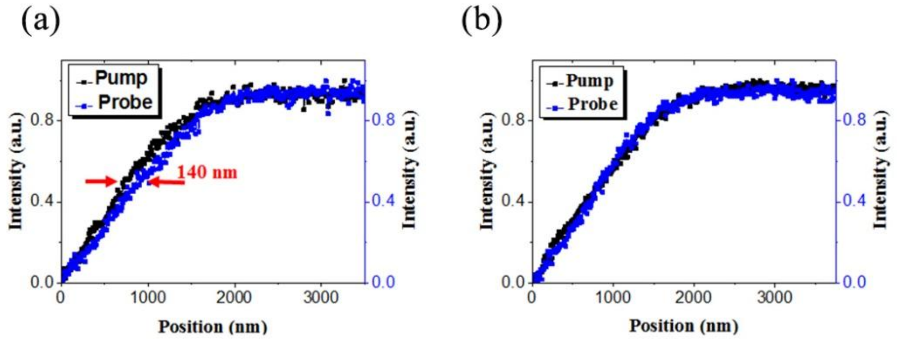

Figure S2 Axial overlapping of pump and probe. (a) Offset of 140 nm; (b) Offset of <20 nm.

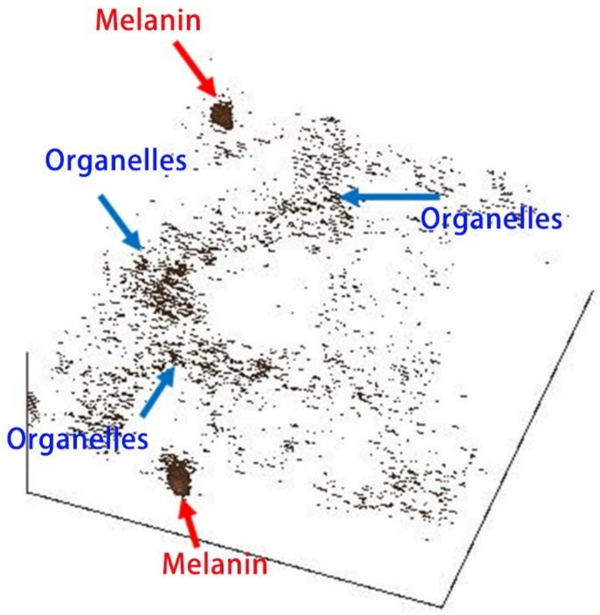

Figure S3 3D image of benign melanoma with low melanin density. Some organelles in the tissue can also be observed. The PT signal from such structures is only 1.1 time larger than the noise level.

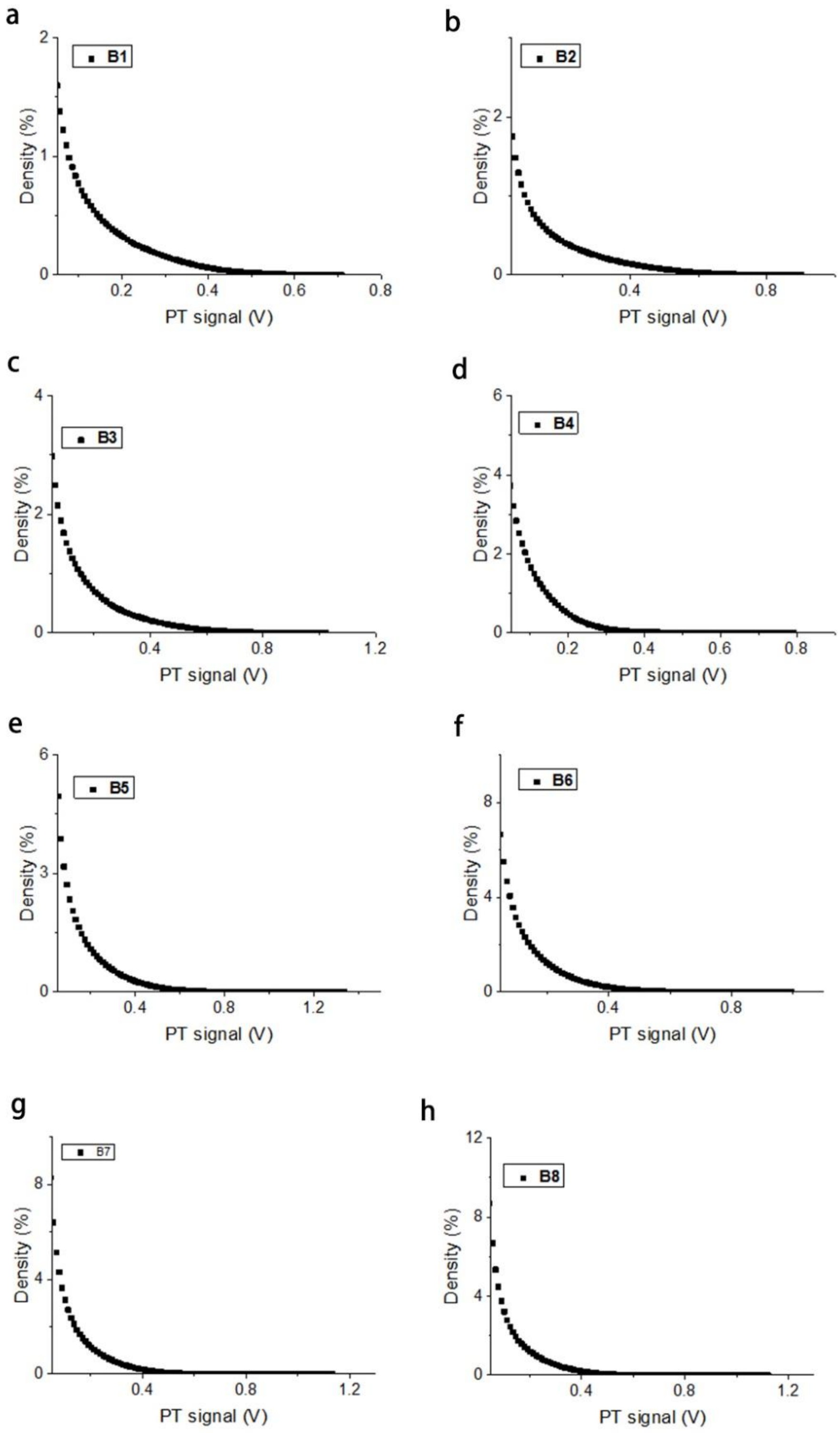

Figure S4 PT signal distribution in 3D images of benign melanoma.

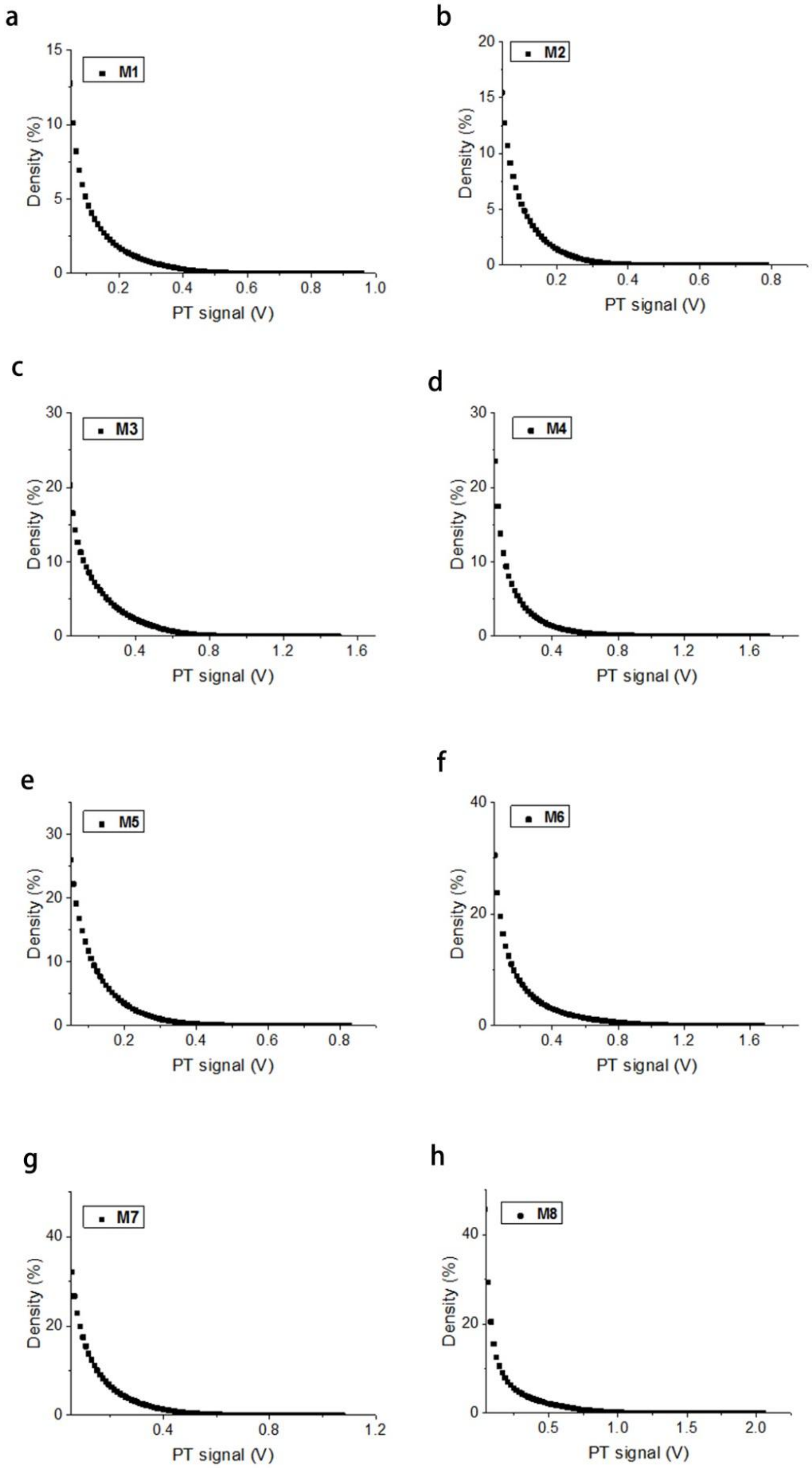

Figure S5 PT signal distribution in 3D images of malignant melanoma.

Threshold	Start box size	Min box size	Box division factor	Number of translation
1-255	24	6	1.2	3

Table S1 Parameters for the fractal dimension calculation with the software ImageJ
